# The Role of Family Medicine Training in Addressing Workforce Challenges in Rural Liberia – Early Implementation Experience

**DOI:** 10.5334/aogh.3249

**Published:** 2021-10-08

**Authors:** Ibrahim Sanoe, Kolu Beyan-Davies, Sarah Anyango, Gerald Ekwen, Jacquelin Pierre, Jessica Farley, Methodius George, Regan H. Marsh, Maxo Luma, David Okiror, Richard Sacra, Rebecca Cook

**Affiliations:** 1 Liberian Ministry of Health, JJ Dossen Hospital, LR; 2 Partners in Health Liberia, JJ Dossen Hospital, LR; 3 Partners in Health Liberia, LR; 4 Maryland County Health Team, JJ Dossen Hospital, LR; 5 Brigham & Women’s Hospital, Harvard Medical School, US; 6 Partners In Health, LR; 7 ELWA Hospital, LCPS, LR; 8 ELWA Hospital, LR; 9 University of Massachusetts Medical School, LR; 10 Massachusetts General Hospital/Harvard Medical School, LR

## Abstract

**Background:**

Liberia has a severe shortage in the health workforce, which is amplified in rural areas. Many talented Liberians leave the country for post-graduate education; those physicians who do stay are concentrated in Monrovia.

**Objective:**

We initiated a family medicine specialty training program (FMSTP) to increase the number of well-trained physicians who have the knowledge, skills, and commitment to meet the health needs of the Liberian people.

**Methods:**

The Liberian College of Physicians and Surgeons (LCPS) family medicine program is a three-year post-graduate course that follows the West African College of Physician (WACP) curriculum. The program has a longitudinal rural training component supported by Partners in Health in Maryland county, where residents gain experience in a remote and under-served region. The program is evaluated through resident evaluations and ultimately bench-marked by accreditation and exam pass rates.

**Findings:**

The FMSTP commenced in July 2017, and the first rural rotation was in January 2018. To-date 13 residents have completed a total of 43 rotations in Maryland. Residents surveyed highly rated the faculty and their rural rotations. They identify more hands-on involvement in patient care, exposure to community health, and one-on-one time with faculty as the greatest assets of the rural training experience. Accreditation from the WACP was granted in December 2018. One of the graduating residents from the first class in 2020 is now serving as the first Liberian family medicine specialist in Maryland County.

**Discussion:**

Investing in a strong rural training component in our FMSTP has not only strengthened the program but has also built the infrastructure to establish our rural site as an attractive teaching hospital for intern doctors and nursing students. As the program continues to grow, success will be measured by the proportion of Liberian medical students entering the family medicine training program, retention of family medicine physicians in rural areas, and ultimately progress towards universal health coverage (UHC).

## Background

Liberia’s health system suffered the dual burden of a prolonged civil war followed by the devastating Ebola epidemic. In 2015, due to significant international migration of health workers during the civil war [1] and the tragic deaths of an estimated 8% of the health workforce from Ebola, Liberia was left with a health workforce density of less than 3.7 per 10,000 population [2]. It was estimated that by 2030, there would be a shortage of 2,700 healthcare workers, and that training programs were not on pace with population growth. As the Ebola epidemic subsided, Liberia’s Ministry of Health implemented programs to not only return to pre-Ebola service levels, but also to build a stronger system that could withstand future challenges. These efforts culminated in the launch of the country’s *Investment Plan for Building a Resilient Health System*, including a national Health Workforce Program (HWP). The resulting HWP Strategy proposed specific interventions across cadres to develop a “fit-for-purpose, productive, and motivated health workforce [3].”

In line with its previous health workforce investments in Africa, such as the Medical/Nursing Education Partnership Initiatives and the Human Resources for Health program in Rwanda, the Health Resources and Services Administration (HRSA), along with support from the President’s Emergency Fund for AIDS Relief (PEPFAR) joined a consortium of donors to support Liberia’s HWP. This investment would create a strong foundation to benefit all health services, and its particular importance for HIV outcomes was clear: in 2016, just 12% of health facilities had at least one staff trained to conduct HIV counseling and testing, and similarly just 12% had at least one staff trained to prescribe and manage Anti-Retroviral Therapy (ART) [4].

In LMICs globally, rural areas experience the greatest health workforce deficits: two-thirds of the population is based in rural areas versus only one-third of all physicians, nurses, and midwives [5]. In Liberia, training for health workers across all cadres is concentrated in Monrovia and other urban areas, and most graduates remain in cities to practice in hospitals and clinics, perpetuating a deficit of health workforce in rural areas. This results in costly travel or the inability to access health care for rural populations. While nearly half of the population of Liberia lives in rural areas, there are marked disparities in health outcomes between urban and rural areas: antenatal coverage is 57.2% in rural versus 86.4% in urban, measles vaccine coverage is 59.6% in rural versus 81.7% in urban areas, and chronic malnutrition is present in 41.5% of children under 5 years in rural Liberia, compared to 29.1% of those in urban areas [6]. Nearly 70% of patients enrolled in ART for HIV are in Monrovia [7].

Family Medicine has played and continues to play a key role in primary health care (PHC) and Universal Health Coverage (UHC) in healthcare delivery in Africa. As a first contact physician who is knowledgeable and skillful in all the major specialties at breadth, a family doctor is expected to improve quality of PHC, improve patient satisfaction and continuity of care, provide comprehensive specialist care at primary hospital level, improve preventive care, develop robust PHC, and participate in transformation of the health system. Family medicine is a developing specialty in sub-Saharan Africa (SSA); the earliest programs were started in South Africa and Nigeria and later extended to East Africa, Ghana, and other countries throughout SSA [8]. While there is heterogeneity in both the training and practice of family medicine across countries, efforts have been made to identify overarching definitions of family medicine, most notably through the Rustenburg statement of consensus on family medicine in Africa in 2009 [9]. The statement contains a thirteen-point list that emphasizes clinical leadership in primary healthcare settings, person-centered care, comprehensive clinical skills appropriate for the population served, and adaptability in low-resource settings [10]. Family Medicine in SSA is further differentiated from family medicine in high-income countries by the additional training emphasis on procedural skills in surgery and OB/GYN required in low-resource settings with limited surgical specialists [8]. Strengthening primary care service delivery is foundational to UHC, ensuring quality health care for the community.

Liberia launched its first post-graduate training programs in 2013, which has since graduated over 35 specialists. There are now residency programs in pediatrics, obstetrics and gynecology (OB/GYN), internal medicine (IM), surgery, psychiatry, ophthalmology, and family medicine. The regional accrediting body for family medicine and all specialty programs in Liberia is the West African College of Physicians (WACP), and the in-country accrediting organization is the Liberia College of Physicians and Surgeons (LCPS). Previously, Liberia’s physician training programs had been largely based in Monrovia, meaning that the many young physicians assigned to work independently in rural facilities were often under-prepared for the unique challenges associated with rural health care or refused to take up their posts. Family physicians remain a scarce resource in Liberia to sufficiently take up assignments in county hospitals and health centers in ensuring UHC. It was thus deemed critical to build a rural rotation into the FMSTP to strengthen the rural health workforce.

The family medicine specialty training program (FMSTP) in Liberia started in 2017. The home training institution is Eternal Love Winning Africa (ELWA) Hospital, a faith-based non-profit hospital in the capital city of Monrovia, and the rural training site is Maryland County, in collaboration with the Maryland County Health Team and Partners in Health (PIH). Faculty from both ELWA and PIH collaborated in the curriculum design and program development of the FMSTP, with PIH taking the responsibility for the rural training component. Residents complete required rotations in Maryland County at JJ Dossen Hospital and Pleebo Health Center, Ministry of Health facilities supported by PIH.

## Methods

In partnership with the Maryland County Health Team and JJ Dossen hospital leadership, PIH has developed a rural physician training platform and complementary curriculum that focuses on developing key skills in rural and primary care service delivery with a focus on training family medicine physicians. The PIH-supported rural training component in Maryland County was developed in response to the need to improve physician workforce and health outcomes in rural Liberia. It provides trainees with exposure to innovative rural health programs as well as the opportunity to learn from experienced clinical personnel, including faculty members in the core clinical disciplines and community health.

**Figure 1:**
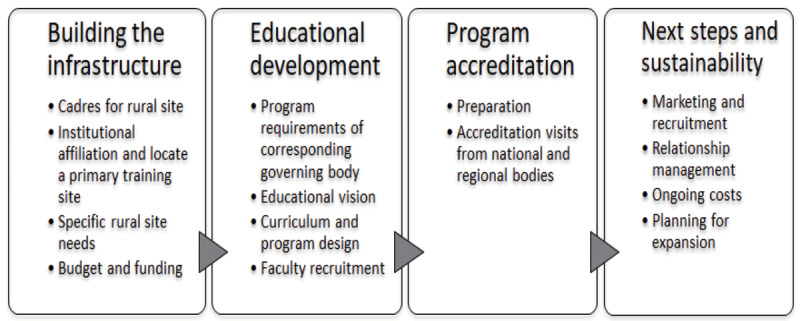
Process for developing rural training platform.

To establish a rural training program in Liberia, we followed an operational framework that included first building the necessary infrastructure for training, developing the educational systems and faculty, completing program accreditation and finally, planning for sustainability (***Figure 1***) [11].

### Building the infrastructure

While it is important to ultimately have trainees exposed to rural healthcare service delivery at every stage of training, the needs and infrastructure for trainees at each level are important considerations in determining which trainees are most appropriate for the rural site to host first. For example, in Maryland County we determined that it would be better to start by hosting residents and intern doctors, because the hospital had adequate clinical specialists and patient volume to support quality clinical training but lacked the additional infrastructure and basic science faculty needed for undergraduate medical education.

There are many infrastructural and operational considerations in operating a rural training program. It is important to consider the extra needs of a training program such as safe, timely transport to rural areas, network coverage and internet access, appropriate length of rotation and opportunities for home leave for trainees’ mental and social wellbeing, on-site secure housing, and availability of food. All these considerations require a detailed assessment to evaluate gaps in the training site and budget for the needed investments.

Aside from direct training costs, we found that it is important to make substantial investments in the healthcare delivery system to be able to reach the standard required for both training accreditation and optimal patient care. Additional considerations specifically for training programs include protected time/salary support for faculty, internet access, access to medical references, physical space for on-call room, library, teaching conferences, housing and food (if included), travel, and safety/security. This budget must be realistic regarding available funding, whether government, private or public-private partnership.

Rural affiliate training sites need academic affiliation to increase credibility of the program, provide academic support and improve the deployment and retention in rural areas. Before hosting any trainee physicians in Maryland County, we established a partnership between JJ Dossen Hospital, PIH, Tubman University, ELWA Hospital, and the Liberian College of Physicians and Surgeons (LCPS), and later extended this academic network by earning accreditation from the WACP.

### Educational development

To develop the program, we had to carefully consider how the program would meet the requirements of governing academic bodies. In Liberia, the post-graduate residency operates under LCPS in Liberia and the WACP regionally. The post-medical school internship year operates under the Liberian Medical and Dental Council (LMDC), while rural service medical officers are assigned by the Ministry of Health (MOH). Each has specific requirements for all training sites in Liberia. The University of Liberia is the governing body for medical school (undergraduate medical education).

While adhering to these external academic and health regulatory bodies is essential, it is also important for the individual training site to develop its own vision. We articulated our own educational philosophy in considering what unique opportunities the rural site offers in the medical education of trainees and how this rural site will fill a niche in medical education. Our training vision is to build the health workforce in an integrated, functioning, patient-centered health system to achieve better patient outcomes. We also created administrative and educational materials and systems for trainees during their rural rotations, including orientation manual, call schedules, rotation evaluations and curriculum materials for each rotation. Tracking systems are also needed for any materials or benefits of the training program for donor reporting. For each specialty area, a board-certified physician specialist faculty is required for training.

### Program accreditation

Assessing a new rural training site may require formal site visits from governing bodies even before a formal assessment or accreditation visit. This process was started with the LCPS in early 2017, prior to the formal accreditation visit. These visits are good opportunities to identify strengths and gaps in training infrastructure, even if accreditation is not granted. The next step for the residency programs is accreditation by a regional body, such as the WACP; the Liberia FMSTP received two-year accreditation from the WACP in December 2018.

### Next steps and sustainability

In many contexts, including Liberia, trainees have choices about training programs, thus it is important to consider advertising and marketing, including highlighting the unique opportunities the rural training program offers and how it can support overall career goals of trainees. Considerations about how to mitigate the hardship of rural practice are also important to consider. Methods of regular communication should be established so that partners are kept informed about programs, progress, staff turnover, and other relevant issues. Currently the costs of our rural training are covered by cost-sharing between the RRHS-HRSA grant, individual donations to PIH-Liberia, and in-kind investments in terms of physical space and some staff from the government of Liberia (GOL). Realistic budgeting is needed to identify strategies for cost-sharing and financing strategies to ensure high-quality training in both urban and rural areas. The long-term sustainability of post-graduate training programs that are largely funded by international donors at the beginning needs to be carefully and strategically planned from inception.

## Program Evaluation

The rural family medicine training program has been continually assessed through end-of-rotation evaluations by both residents and faculty as well as annual program reviews which are covered under PIH clinical research protocol with the University of Liberia IRB. These surveys include both close-ended Likert scale questions and open-ended questions, and also a section for comments. Residents complete evaluations via paper form or electronic survey; data is then de-identified and entered into an electronic database in Microsoft Excel on secure password protected computers. Narrative feedback from residents and faculty has also been collected through regular faculty meetings and a joint faculty and trainee family medicine retreat in 2019. Trainees were informed that de-identified data from these evaluations could be used for program improvement and for dissemination of lessons learned to a wider audience. Measures of essential health service provision at the rural training sites (JJ Dossen Hospital and Pleebo Health Center) have been monitored via District Health Information System-2 (DHIS-2) and clinic-level databases in the HIV program in order to evaluate the impact of training program on universal health coverage. Finally, the outcomes of the program, in terms of graduating residents and successful long-term retention of these physicians, are critical measures of the impact of the training program; however, we are currently early in the training program for these outcomes to be measurable.

## Results

**Figure 2:**
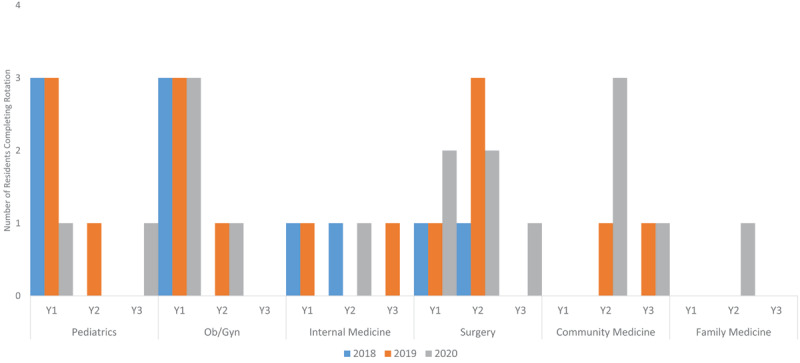
Rural Rotations for FMSTP in Maryland, Liberia 2018–2020.

The Liberian FMSTP commenced in July 2017 and the first rural rotations in Maryland were initiated in January 2018. Each resident rotates a minimum of 8 weeks/year in Maryland during each year of residency training. During the first and second years (Y1 and Y2), rotations include core clinical rotations: Pediatrics, Ob/Gyn, Surgery, and Internal Medicine. During these rotations, residents rotate primarily on the inpatient wards at JJ Dossen, the county referral hospital, but also participate in specialty clinics (chronic disease clinic, gynecology, high-risk obstetrics, and surgery clinics). Those on their pediatric and Ob/Gyn rotations also make visits to the largest primary health center in the county. During the second or third year (Y2 or Y3), residents also complete a unique rotation in community health where they serve as the primary doctor at the largest-volume health center in the county (Pleebo Health Center). To-date, 13 family medicine residents have trained in Maryland completing a total of 43 individual rotations (***Figure 2***).

Resident teaching includes a morning report, clinical teaching rounds, and didactic sessions with consultants. Residents are also required to develop as medical educators and give presentations with interns and mid-level providers to help strengthen their teaching and mentoring skills. In their community health rotation, residents serve as the main doctor at Pleebo Health Center, the highest volume health center in the county, located 45 minutes away from the referral hospital. During this rotation they get experience practicing the breadth of family medicine, referring complex cases, conducting home visits for vulnerable patients, and engaging with the community in health prevention. Starting in 2020, we also hosted a resident on the family medicine rotation during which they rotated through various outpatient clinics and conducted regular home visits for complex and palliative care patients.

Residents completed an evaluation at the end of each rotation in Maryland that included the Likert scale for reviewing the academic, operational and system aspects of the program (***Table 1***).

**Table 1:** Family Medicine Residents’ Review of Academic Rotations in Rural Liberia.


	OB/GYN	PEDIATRICS	SURGERY	INTERNAL MEDICINE	COMMUNITY HEALTH

The Attending made the goals and learning objectives clear at the start of the rotation	4.50	4.60	4.33	4.67	5

The Attending communicated complex information in a way that was clear and understandable	4.33	4.80	4.50	4.67	4

The Attending allowed me to participate in clinical situations in a way that facilitated learning	4.50	4.60	4.83	4.67	4

The Attending was responsive to questions from residents	4.67	4.80	4.83	4.67	5

The Attending and staff in my program were interested in my residency education	4.33	4.80	4.67	4.67	4

The Attending and staff provided sufficient supervision	4.50	4.20	4.50	4.33	4

The Attending and staff provided sufficient instruction	4.17	4.40	4.33	4.67	4

The Attending effectively created an environment of scholarship and inquiry	4.60	4.00	4.40	4.33	4

This program provided an environment where residents could raise problems or concerns without fear of intimidation or retaliation	4.00	4.40	4.00	4.33	4

I am satisfied with this program’s process to deal confidentially with problems or concerns residents might have	4.00	4.20	4.17	4.33	3

I am satisfied with the opportunities this program provided for me to participate in research and/or scholarly activities	4.17	4.20	3.83	4.33	4

I learned new skills that will be valuable in my future clinical practice	4.33	4.40	4.33	4.33	5

I had sufficient opportunities to practice new skills during this rotation	4.17	4.00	4.50	3.67	4

I would recommend this rotation to fellow Residents	4.17	4.60	4.50	4.00	5


1 = strongly disagree 2 = disagree 3 = neither agree nor disagree 4 = agree 5 = strongly agree. N = 13 family medicine residents, completed a total of 24 rotation evaluations (2018–2020).

The residents uniformly had positive reviews of the faculty on rotations noting that faculty consistently set learning goals, provided teaching and adequate supervision, and created an environment of scholarship and inquiry. All residents stated at the end of their rotation that they would recommend or highly recommend the rotation to other residents.

Resident feedback on program and systems supports, such as housing, transportation, internet and hospital systems, were generally rated as fair or good (***Table 2***), although residents consistently made additional suggestions for improvement in operational and hospital systems to improve the training experience.

**Table 2:** Family Medicine Resident Evaluation on System Support in Rural Training Program.


CATEGORY	1 = POOR	2 = FAIR	3 = GOOD	4 = EXCELLENT	AVERAGE SCORE

The quality of my housing arrangements	0%	25%	58%	17%	**2.92**

The quality of the food provided	0%	40%	40%	20%	**2.80**

Transportation	0%	9%	45%	45%	**3.36**

Level of financial support	17%	0%	58%	25%	**2.92**

Hospital response to safety	33%	33%	33%	0%	**2.00**

Quality of internet	8%	75%	17%	0%	**2.08**

Length of rotation	0%	25%	58%	17%	**2.92**

Opportunities for social interaction	8%	25%	42%	25%	**2.83**

Opportunities for professional interaction	0%	8%	42%	50%	**3.42**

Opportunities for training in HIV treatment and prevention	0%	0%	50%	50%	**3.50**

Clinical resources in the hospital unit(s) where I worked	8%	25%	50%	17%	**2.75**


N = 13 family medicine residents who rotated in Maryland County.

Qualitative feedback on the program was collected through open-ended questions in the evaluations along with a residency program retreat in 2019 and regular check-ins with the trainees. Themes from this feedback are discussed below.

### Increased exposure and confidence in patient care

Many residents noted gains in new procedural skills and more confidence in skills they had previously. One resident stated that the rotations in Harper

“further strengthened my ability to comfortably manage surgical emergencies like acute appendicitis, gastric/bowel perforations, inguinal hernia reduction and repair of hernias, chest tube insertion, and other minor surgical procedures like suturing of lacerations. [My OB/GYN rotation] further strengthened my ability to comfortably manage obstetric emergencies like pre-eclampsia/eclampsia, post-partum hemorrhage, and Emergency cesarean sections.”

On internal medicine rotations residents highlighted new skills in conducting and interpreting EKGs and bedside ultrasounds, while in pediatrics they particularly highlighted gains in competence in managing neonates and children with severe acute malnutrition, two of the leading causes of childhood deaths in Liberia. There was an increased confidence in taking care of critical and complex patients. One resident summarized that through their rotation in Maryland, they “improved on the skills required to approach and deal with critical care needed to handle patients who are in critical conditions.” Several residents highlighted important growth in clinical management and decision-making with limited resources as an important asset of rural training.

### Accessibility and close mentorship from rural family medicine faculty

Residents frequently highlighted close relationships with faculty as one of the main assets of the rural training program. One resident summarized it well—that the best aspect of the rotation was the “one-on-one clinical/surgical skills and directed oversight [from the attending] and detailed work plan on goals to be achieved from day one.”

### Transformative experience in community health

Aside from skills and medical knowledge gained, residents also reported important growth in communication, counselling and understanding of the patients and community they serve. One of the residents explained that they gained “Improved level of patient and physician interaction in understanding patient feelings and needs” that they would not have gained at an urban teaching hospital. Another wrote, “Communicating with my patients in a simple way that they will understand; speaking Liberian English, and or using sign language or using an interpreter to communicate well.” With particular reference about what they had learned about HIV, one resident cited improved “Ability to recognize early signs of the disease, uncommon symptoms and to deal with emotional consequences of the disease on the part of the patient and family.” Another resident highlighted novel experiences such as “Working with youth groups to understand the needs and challenges of rural youth and the opportunity to visit the patient at home.”

### Areas for Improvement: Living and working environment for trainees and health systems

In their open-ended comments, residents suggested specific improvements to housing (need for more recreation options and space, improvement in security at the hospital), food (hiring a cook for trainee doctors on-site, more options and choice), internet, and safety. In addition, residents made constructive suggestions on how the hospital system could be improved to enhance training and patient care, including highlighting the needs for improved staffing and emergency response in caring for critical patients, and systems improvements such as regular morbidity and mortality conferences.

### Achieving Accreditation

Two-year accreditation from the West African College of Physicians was granted in December 2018. Leading up to this accreditation, there was intensive preparation with the faculty of Family Medicine at ELWA along with the LCPS to ensure that all the supporting documents, including resident rotation calendars, didactic schedules, and training and procedure logs were in-place. In addition, faculty ensured standardization of the learning goals and objectives for each rotation offered in the program, and updated curriculum vitae and licensing documented for all the faculty. Three fellows from the WACP faculty of family medicine made a day visit to Harper for the accreditation visit, toured the facility, met with faculty and hospital leadership and interviewed residents. While earning the two-year accreditation was a milestone, the accreditors also gave important constructive suggestions including the need for important systems improvements for the health facility including improving the medical records and waste management systems which PIH has been gradually working with the MOH to improve.

### Impact of Rural Family Medicine Rotation on the Health System in Maryland County

The investment in faculty, health systems, training, and physical infrastructure in order to host an accredited FMSTP has had an important impact on the health system: It has expanded access to additional trainee physicians and directly improved patient outcomes. Because of the family medicine training program and the commitment by PIH to place faculty at JJ Dossen hospital, the reputation of the hospital as a teaching hospital has grown.

**Figure 3:**
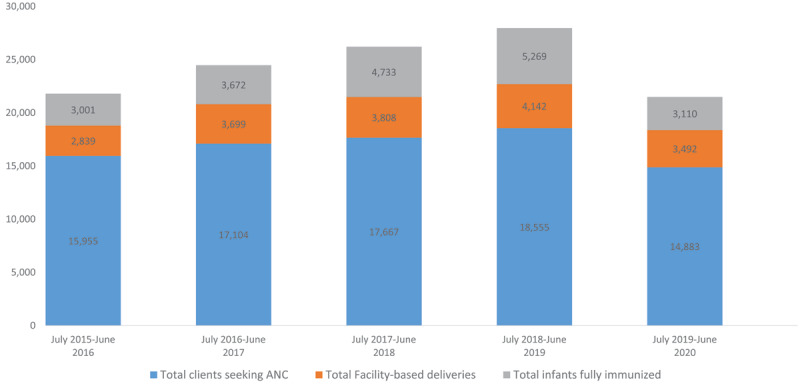
Maternal and Child Health Indicators in Maryland County, July 2015–June 2020.

**Figure 4:**
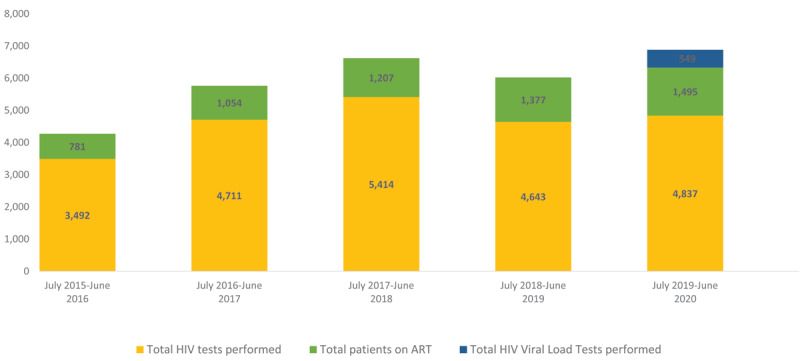
HIV Performance Indicators at JJ Dossen Hospital and Pleebo Health Center, 2015–2020.

Intern doctors and EMONC doctors started to be assigned to JJ Dossen as well, when historically the LMDC and MOH had not sent trainee physicians to such remote county hospitals. Since the initiation of FMSTP, in addition to family medicine residents, JJ Dossen Hospital has trained four EMONC doctors and ten interns. Early investments in FMSTP have helped developed a broader rural training platform for physicians. In addition, significant investments have been made in the health system in Maryland that have led to improved maternal and child health outcomes (***Figure 3***) and HIV program indicators (***Figure 4***). While there is an exception in the progress in MCH utilization indicators in 2020, largely due to coronavirus pandemic, our findings demonstrate that commitment to excellence in rural physician training is aligned with improved health care coverage for the population through investment, not just in the trainees but in the health care delivery system.

## Discussion

Residents choose the FMSTP for more hands-on experience and breadth of clinical skills and experience than they can get in more focused specialty programs at the national referral hospital. Applicants to the FMSTP voice the desire to be well-trained to meet the breadth and depth of population needs based on current health workforce realities in Liberia; about half have served as GPs for several years prior to the specialty program while the other half came directly from internship and full licensure.

Teaching in the FMSTP focuses on history, physical examination, good communication, and proper follow-up, with rigorous hands-on training in the operating room alongside clinical and community health QI projects pertinent to Liberia. Trainees work hard and learn from one another, from dedicated faculty, and importantly from the patients and communities they treat and serve.

A major challenge in the Liberian medical system is recruiting physicians into rural service after training. This challenge is echoed throughout sub-Saharan Africa (SSA). Medical schools are frequently located in the capital or other major cities, and medical students are more likely to come from urban areas as well. Multiple studies across several SSA countries reveal homogeneity in concerns about pursuing rural service: poor health facility equipment and infrastructure, lack of supervision and management support, limited career advancement opportunities, spartan living conditions, cultural and language differences, and distance from family [12]. However, there are also predictors and motivators for rural practice. Rurally based physicians in northern Ghana cited the opportunity to gain additional clinical skills and their previous experiences with rural service during their medical education as the main decision-making factors [13]. Similarly, a study of medical students from two medical schools in Ghana, one with a traditional curriculum and one with a problem-based learning and community-based education component, showed that students in the traditional program were 80% less likely to state that their training adequately prepared them for rural service [13]. In an eight-country survey of final-year medical and nursing students in SSA and Asia, predictive factors for rural service included previous exposure to rural settings and altruistic career values [14]. Among junior doctors in Sudan supervision, bonus pay, and facility equipment were the strongest incentives for rural service [15].

The FMSTP was deliberately designed to address many of the common barriers to rural physician service and provide evidence that it is feasible and effective to develop high-quality training in rural areas. Residents in our training program note that during their rural training experience, they perform many clinical tasks, including surgery, sooner and more independently than at larger urban referral hospitals, and have more one-on-one contact time with consultants for teaching. This is consistent with a trend identified in the literature of the enhanced exposure for clinical skill-building as a draw for rural service. Residents’ experience in rural Liberia, particularly on their community health rotations, has transformed their perspective on health care delivery in rural areas, made them more aware of the tremendous unmet health needs in rural areas, and expanded their horizons to what medical practice as a family physician can mean.

Many investments in JJ Dossen Hospital and Pleebo Health Center were made to address some of the challenges identified other studies regarding rural training and service, including on-site housing, adequate supervision by well-trained faculty, investment in health facilities in terms of supplies and equipment, basic internet (although continued improvements are needed), and establishing a dedicated medical library. We found that close collaboration with the hospital leadership along with national training bodies has been key to the success of the program. In addition, planning for and securing funding for the operational aspects of the training program (adequate housing, feeding, transportation) has often been more challenging than developing the academic components of the program. Financial sustainability is a major challenge across the physician health workforce programs in Liberia; much of the funding for post-graduate physician training in Liberia has been provided by international donors with short-term (three to five year) grants post-Ebola, while the typical time frame for developing a sustained residency training program fully run by experienced local faculty is at least 10 years. A longer-term vision and commitment from the international community should be coupled with robust joint planning—not only with the ministry of health but also the ministry of finance and administration—to ensure sustainability of training programs and absorption of trained specialists into the health workforce.

The increasing interest from trainee physicians in coming to Maryland is stretching the current capacity of the program. To address this challenge, we plan to continue to develop regular and systematic evaluation of the training program, including 360 evaluations for both trainees and faculty. Synchronization of resident, intern, and EMONC rotations is needed to avoid overcrowding of clinical services and faculty at any given time. Additionally, there is a need for continued investment for expansion in physical space, staff, and supplies for long-term sustainability. Faculty development and career pathways are also important in further mentorship and retention of faculty; this includes incorporation of rural faculty into national academic colleges and societies, participation in training conferences and seminars, and support for scholarly pursuits.

Despite the challenges, the family medicine residents have demonstrated flexibility and understanding, and have overall had a tremendously positive perspective about their training in Maryland. They have served as ambassadors that are not only making family medicine one of the most highly enrolled post-graduate training programs, but also recommending the hospital to interns and EMONC doctors. It will take time to show the full impact of rural exposure during the FMSTP on rural practice in Liberia. However, one of the first two family medicine residents to graduate is now working full time at JJ Dossen, and as of August 1, 2021 will assume the role of Medical Director of JJ Dossen Hospital, which is a promising step in sustainability.

## Conclusion

Although the FMSTP has only graduated its first class at the time of this publication, the program has started to strengthen the rural health workforce, with one of the first graduates serving in rural Liberia. We hope that the operational framework and budget details provided will assist other nascent rural training programs. Early experiences from Liberia echo the findings of a larger study on family medicine physicians in 2014, which noted that family medicine physicians can play an important role in health care system in Africa [8]. In Liberia, the FMSTP is still young, and exists in a wider ongoing effort of health workforce strengthening and rebuilding led by the MOH. In parallel to the progress described in this paper on development of the clinical training program, efforts to define and codify the role of family medicine in the Liberian public health system are essential as the program develops. Flinkenflögel, et al. note that across SSA “a lack of clarity on the scope and practice of FM among policymakers often leads to the discipline not being fully integrated into health systems.” Establishing a clear salary band and promotion policy for specialist physicians in the MOH, including those in family medicine, are critical steps towards long-term retention. There is now a Society of Family Medicine Physicians in Liberia to further advocate and guide policy on the role FM physicians play within the health system. The integration of the family medicine physician into the local health system is an important contribution to improving the health workforce in rural Liberia.

## Abbreviations

Emergency Obstetric & Neonatal Care (EmONC)

Eternal Love Winning Africa (ELWA)

Family Medicine (FM)

Family Medicine Specialty Training Program (FMSTP)

Human Immunodeficiency Virus (HIV)

Liberian College of Physicians and Surgeons (LCPS)

Liberian Medical and Dental Council (LMDC)

Maternal and Child Health (MCH)

Ministry of Health (MOH)

Non-Communicable Disease (NCD)

Partners In Health (PIH)

Primary Health Care (PHC)

Responsive and Resilient Health System (RRHS)

Sub-Saharan Africa (SSA)

World Health Organization (WHO)

Universal Health Care (UHC)
